# CaMKII content affects contractile, but not mitochondrial, characteristics in regenerating skeletal muscle

**DOI:** 10.1186/s12899-014-0007-z

**Published:** 2014-12-17

**Authors:** Wouter Eilers, Richard T Jaspers, Arnold de Haan, Céline Ferrié, Paola Valdivieso, Martin Flück

**Affiliations:** Institute for Biomedical Research into Human Movement and Health, Manchester Metropolitan University, John Dalton Building, Oxford Road, M1 5GD Manchester, United Kingdom; Laboratory for Myology, MOVE Research Institute Amsterdam, Faculty of Human Movement Sciences, VU University Amsterdam, Van der Boechorststraat 7, 1081 BT Amsterdam, The Netherlands; Laboratory for Muscle Plasticity, Department of Orthopaedics, University of Zurich, Balgrist University Hospital, Forchstrasse 340, 8008 Zurich, Switzerland

**Keywords:** CaMKII, Skeletal muscle, Plasticity, Excitation, Contraction, Mitochondria

## Abstract

**Background:**

The multi-meric calcium/calmodulin-dependent protein kinase II (CaMKII) is the main CaMK in skeletal muscle and its expression increases with endurance training. CaMK family members are implicated in contraction-induced regulation of calcium handling, fast myosin type IIA expression and mitochondrial biogenesis. The objective of this study was to investigate the role of an increased CaMKII content for the expression of the contractile and mitochondrial phenotype *in vivo*. Towards this end we attempted to co-express alpha- and beta-CaMKII isoforms in skeletal muscle and characterised the effect on the contractile and mitochondrial phenotype.

**Results:**

Fast-twitch muscle *m. gastrocnemius* (GM) and slow-twitch muscle *m. soleus* (SOL) of the right leg of 3-month old rats were transfected via electro-transfer of injected expression plasmids for native α/β CaMKII. Effects were identified from the comparison to control-transfected muscles of the contralateral leg and non-transfected muscles. α/β CaMKII content in muscle fibres was 4-5-fold increased 7 days after transfection. The transfection rate was more pronounced in SOL than GM muscle (i.e. 12.6 vs. 3.5%). The overexpressed α/β CaMKII was functional as shown through increased threonine 287 phosphorylation of β-CaMKII after isometric exercise and down-regulated transcripts COXI, COXIV, SDHB after high-intensity exercise *in situ*. α/β CaMKII overexpression under normal cage activity accelerated excitation-contraction coupling and relaxation in SOL muscle in association with increased SERCA2, ANXV and fast myosin type IIA/X content but did not affect mitochondrial protein content*.* These effects were observed on a background of regenerating muscle fibres.

**Conclusion:**

Elevated CaMKII content promotes a slow-to-fast type fibre shift in regenerating muscle but is not sufficient to stimulate mitochondrial biogenesis in the absence of an endurance stimulus.

## Background

Repeated muscle contractions rely on motoneuron-driven variations in sarcoplasmic calcium though ryanodine receptor-mediated release of calcium from the sarcoplasmic reticulum (SR) and the subsequent re-uptake of calcium via SERCA channels [1]. The observed magnitude of the rise in sarcoplasmic calcium varies between slow and fast muscle types, which suggests it may control the contractile characteristics of muscle fibres [2]. However, this relationship is not fixed and muscle fibre makeup in SR and contractile proteins demonstrates a certain degree of plasticity in response to contractile stimuli [3]. An important feature of repeated muscle work (i.e. endurance training) is the specific increase in mitochondrial content; reflecting a compensatory strategy to meet the energy demand of fibres undergoing frequent rounds of actin and myosin cross-bridge cycling [4]. In the rat, the mitochondrial adaptations of exercised skeletal muscle are associated with a transition of myosin isoform expression towards a slow-twitch phenotype [5,6]. The observed transformation of muscle fibre types is associated with a chronic rise in sarcoplasmic calcium [7-9], which implicates calcium-dependent biochemical pathways in the regulation of muscle plasticity.

The calcium/calmodulin-dependent phosphatase calcineurin and calcium/calmodulin-dependent kinases (CaMK) are important transducers of calcium signals towards gene expression [2,10]. The calcineurin-mediated pathway has been shown to regulate expression of slow fibre type-related myofibrillar proteins [11-13] and affects mitochondrial gene expression although calcineurin does not appear required for exercise-induced mitochondrial biogenesis [14-16]. By contrast, CaMK activation has been implied to regulate mitochondrial biogenesis [9,17,18], type IIA myosin heavy chain (MHCIIA) expression [19,20] and calcium re-uptake into the SR in slow type muscle fibres [21].

However, the current understanding of the physiological role of CaMK holoenzymes in whole muscle is incomplete, as previous studies rely on short-term inhibition studies with pharmacological agents in single muscle fibres [22] or the overexpression of a CaMKIV mutant which is not expressed in skeletal muscle [23] and has lost its calcium-dependent regulation [18]. CaMKII is the main CaMK isoform in skeletal muscle. Importantly, however, the effects of CaMKII on the expression of genes that underlie muscle plasticity have - apart from the study of the glucose transporter GLUT4 [24] - not been addressed.

CaMKII operates as a hetero-meric phospho-transferase which can decode calcium transients through auto-phosphorylation at threonine-287 ([2,25,26]). Threonine-287 phosphorylation of CaMKII is increased after acute exercise in rats and humans [27,28], indicating that CaMKII is part of the signalling pathways integrating the effects of exercise on muscle structure and function (reviewed by [2,29]). These findings emphasize that CaMKII activation is firmly associated with the regulation of the oxidative muscle phenotype with contractile paradigms [2,30]. This is corroborated by the concomitant increase in CaMKII and mitochondrial ATP synthase expression with endurance exercise training [31,32]. However, the question remains to which extent contractile features, as shown by CaMK inhibition in vitro [19,20], depend on CaMKII in vivo, and to which extent downstream effects of CaMKII-mediated calcium sensing would be differently affected between contractile muscle phenotypes [33,34].

We hypothesized that the content of hetero-multimeric CaMKII controls the mitochondrial and contractile phenotype of skeletal muscle and that this would take place in both slow-twitch *m. soleus* (SOL) and fast-twitch *m. gastrocnemius* (GM). We tested this assumption by assessing the effects of the co-overexpression of native alpha- and beta-CaMKII isoforms, with similar substrate specificity and structure as the skeletal muscle CaMKII isoforms [35,36] on selected protein markers of the contractile and mitochondrial phenotype. Additionally, the corresponding transcript response to high-intensity exercise *in situ* was measured along with functional characteristics of the targeted SOL and GM muscles. Because the muscles under investigation show fibre recruitment during self-initiated locomotion [37] we assumed that the effect of CaMKII overexpression would manifest under normal cage activity without an imposed contraction protocol. Control experiments were carried out to quantify the extent of muscle regeneration being associated with the selected electro-transfer method to produce overexpression from injected expression plasmids [38-40].

## Results

### CaMKII overexpression and phosphorylation in skeletal muscle

**Figure 1:**
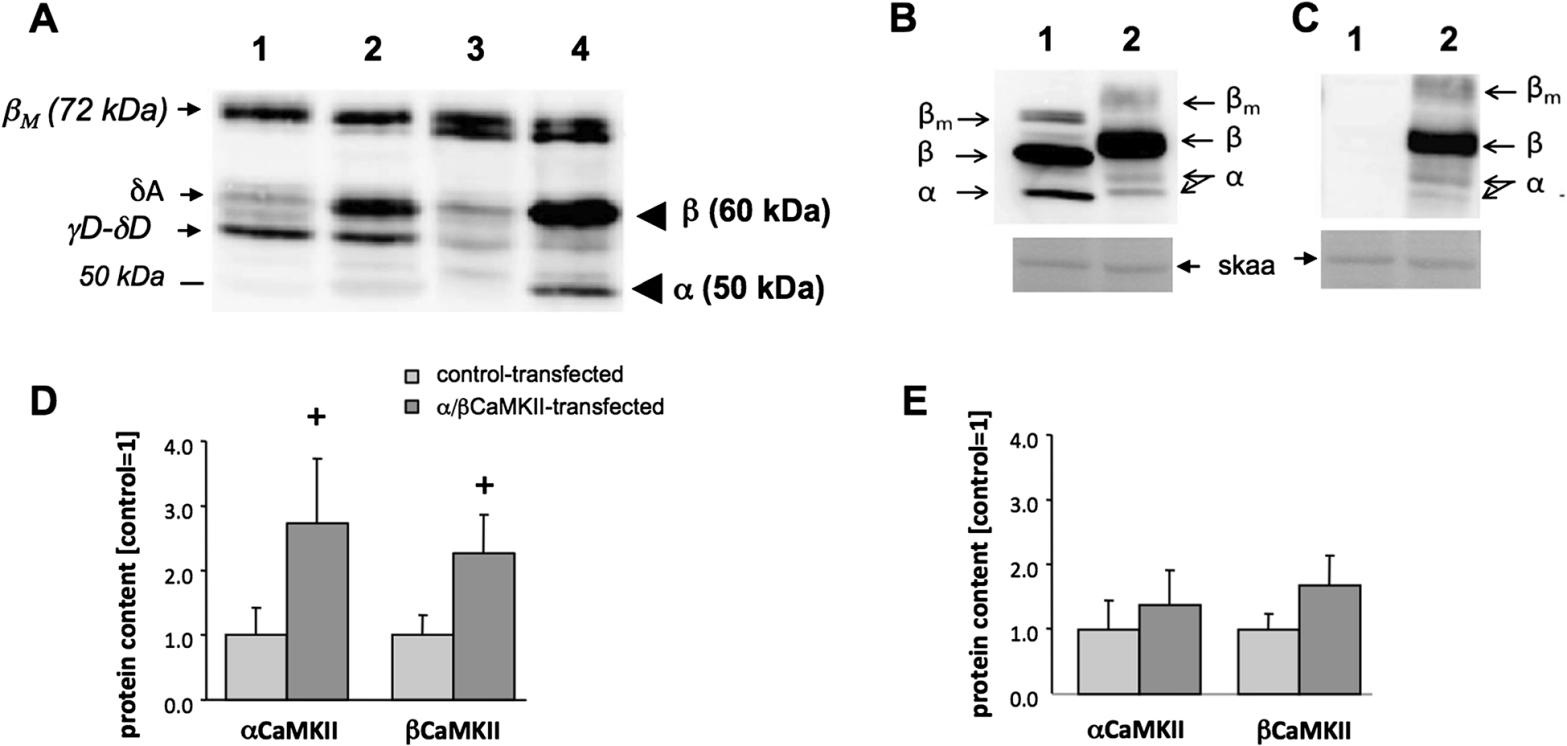
**α/β CaMKII co-overexpression in soleus and gastrocnemius muscle. A)** Immunoblot of homogenate from a control-transfected (1,3) and α/β CaMKII-transfected (2,4) GM (1,2) and SOL (3,4) and muscle pairs after detection with antibody against pan-CaMKII. **B, C)** Immunoblot with pan-CaMKII **(B)** and pThr287-CaMKII antibody **(C)** of in vitro kinase reactions of homogenates from a α/β CaMKII-plasmid injected and porated soleus muscle after incubation in conditions: 1) suppressing (EGTA) and 2) allowing (2, Ca^2+^/CaM) calcium/calmodulin dependent autophosphorylation. Loading controls of the respective Ponceau S-stained membrane visualizing the skeletal α actin band are shown below. Arrows indicate the position of the bands corresponding to Calcium/Calmodulin-inducible kinase II isoforms. α and β correspond to the overexpressed CaMKII isoforms. **D, E)** Bar graph showing the mean + SE of α/β CaMKII protein content in control-porated (light grey filled bar) and porated and α/β CaMKII co-transfected (light grey filled bar) *soleus* **(D)** and gastrocnemius medialis **(E)** muscle. +, 0.05 ≤ p <0.10 vs. control-porated muscle, respectively. n = 4.

α/β-CaMKII-transfection increased protein levels of α- and β-CaMKII isoforms at 50 and 60 kDa, respectively, compared to control-transfected SOL muscle (Figure 1A). *In vitro* experiments demonstrated Ca^2+^/CaM-dependent phosphorylation of the introduced β CaMKII (Figure 1C). Overexpression of α/β CaMKII could be detected in 12.6% of fibres in SOL muscle and at a lower level, i.e. 3.5%, in GM muscle (p = 0.002). α- and β-CaMKII content was increased at the level of total protein in SOL muscle (Figure 1D/E). Fibres in SOL and GM muscle which demonstrated elevated CaMKII expression after the transfection of α/β CaMKII plasmid had 5.3 ± 0.8 and 4.0 ± 0.5 fold increased CaMKII levels, respectively. Mean cross sectional area (MCSA) of the transfected muscle fibres was lower than the non-transfected fibres in the α/β CaMKII-transfected SOL muscle (transfected vs. non-transfected muscle fibre: 2675 ± 132 vs. 3706 ± 86 μm2; p = 0.001).

### α-and β-CaMKII co-overexpression shifts gene expression towards a fast phenotype with enhanced calcium handling

**Figure 2:**
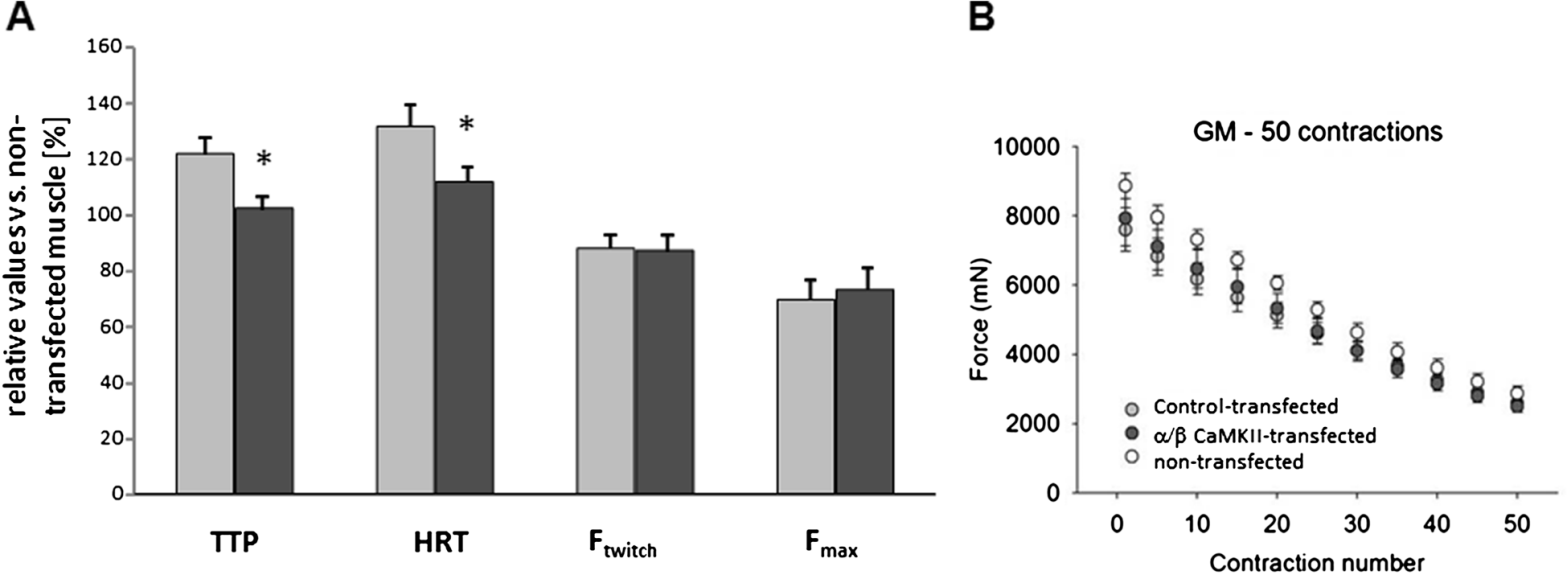
**Contractile effects of α/β CaMKII co-overexpression. A)** Mean + SE of values for the contractile parameters time-to-peak-twitch force (TTP), half-relaxation time (HRT), maximum twitch force (F_twitch_) and maximum tetanic force (F_max_) for the SOL and GM muscle combined being subjected to control-transfection (light grey filled bar) or α/β CaMKII-transfection (dark grey filled bar). Values reflect values relative to the average seen in non-transfected muscle (n = 7). *, p ≤0.05 vs. control-transfected muscle. Repeated ANOVA with post hoc test of Fisher (n = 11). **B)** Scatter plot of mean + SE of force values of fifty paced tetanic contractions in non-transfected, control-transfected, and α/β CaMKII-transfected GM muscle. n = 6-9 per group.

Factorial analysis of the paired data revealed a main effect (p < 0.05) of the co-overexpression of the native α- and β-CaMKII isoforms on the contractile parameters time-to-peak-twitch force (-16%) and half-relaxation-time (-15%) for the combined data from SOL and GM muscle (Figure 2). In SOL muscle alone, a trend for a shortened half-relaxation-time was identified. Maximum twitch force and maximum tetanic force, and fatigability of tetanic force, did not differ between control- and α/β CaMKII-transfected GM and SOL.

**Figure 3:**
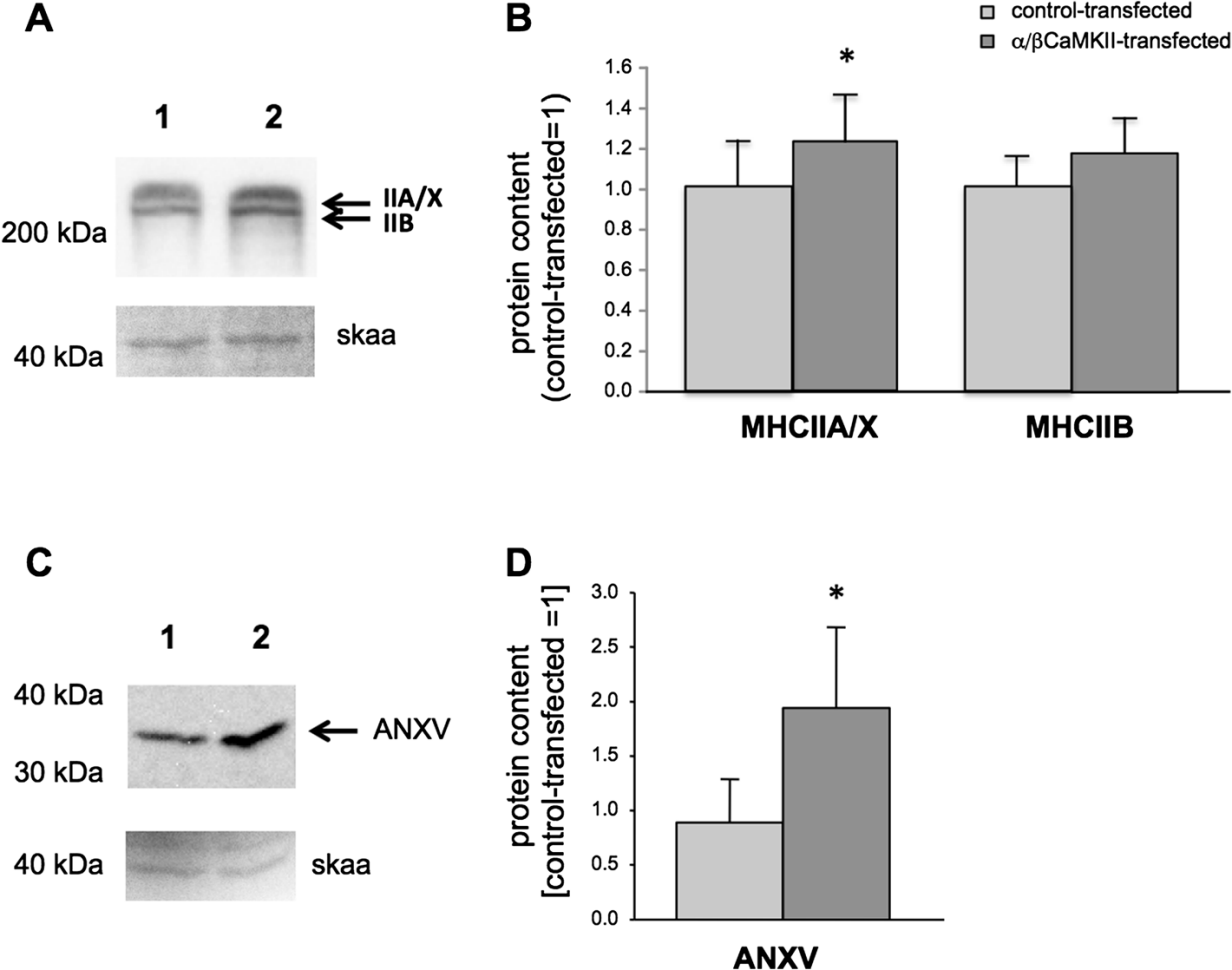
**α/β CaMKII co-overexpression increases MHCIIA/X but not mitochondrial protein. A, C)** Immunodetection of fast type myosin heavy chain (MHCII) **(A)** and ANXV **(C)** in homogenate from a control-transfected (1) and α/β CaMKII-transfected (2) SOL muscle pair. Below loading control visualizing skeletal alpha actin (skaa). **B, D)** Bar graph visualising the mean + SE of MHCA/IIX and MHC IIB **(B)** and ANXV protein content **(D)** in control-transfected (light grey filled bar) and α/β CaMKII-transfected (dark grey filled bar) SOL muscle (n = 9). *, p ≤0.05 vs. control-transfected (Wilcoxon test).

**Figure 4:**
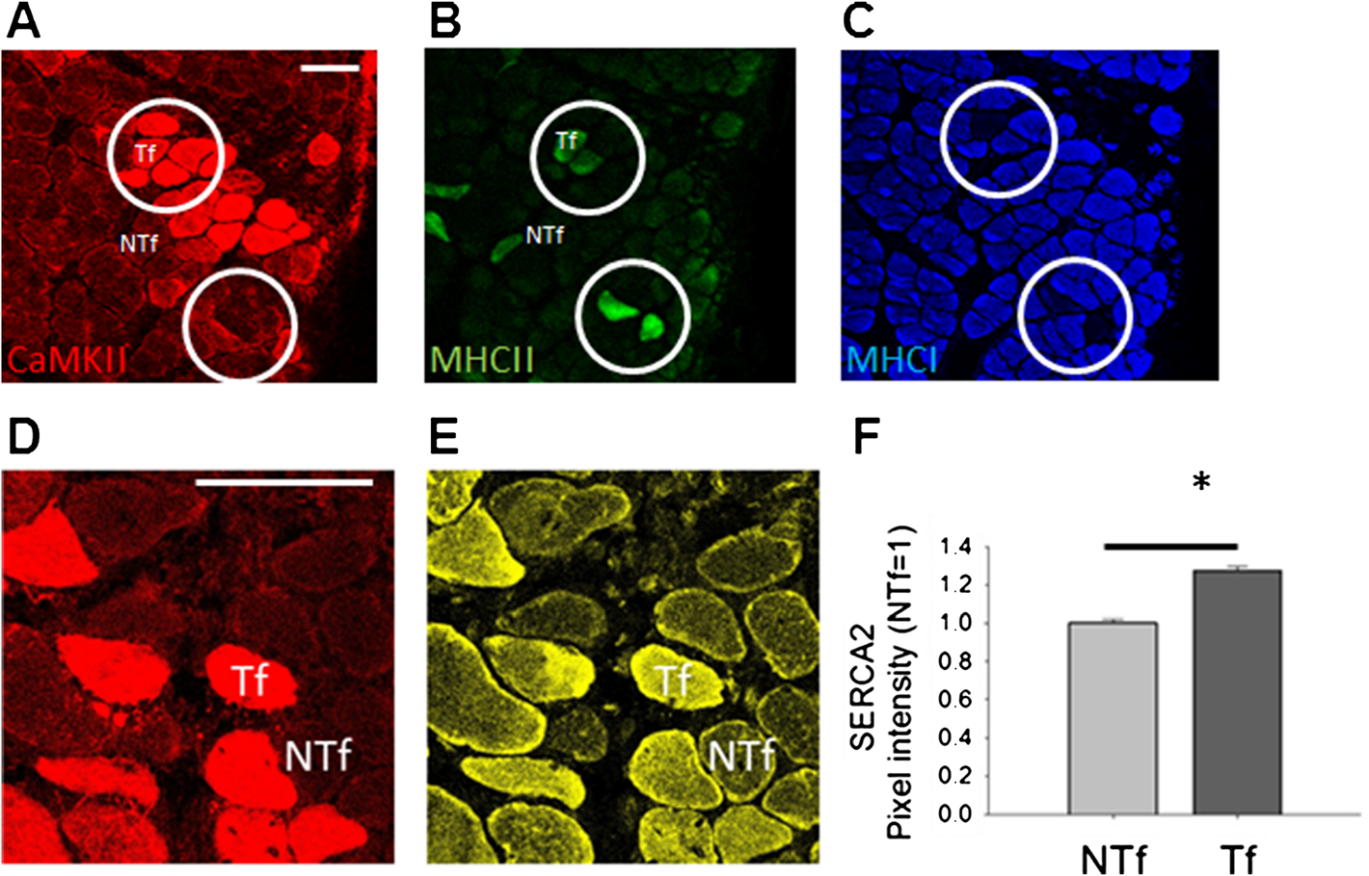
**α/β CaMKII co-overexpression in relation to MHC and SERCA2 expression. A-C)** Microscopic field of α/β CaMKII-transfected SOL muscle stained for CaMKII **(A, red)**, fast MyHCII **(B, green)** and slow MHCI **(C, blue)**. Corresponding areas are circled. **D, E)** Microscopic field of an α/β CaMKII-transfected SOL muscle after staining for CaMKII **(red, D)** and SERCA2 **(yellow, E).** **F)** Bar graph visualizing the mean + SE of SERCA2 levels in transfected (Tf, n = 276) and non-transfected (NTf, n = 221) muscle fibers. *, p ≤0.001.

We assessed the effect of α/β CaMKII overexpression on the levels of proteins associated with the acceleration of muscle contraction (i.e. MHCIIA/IIX and IIB) and relaxation (SERCA2, ANXV; [4, 67]). This analysis was limited to SOL, because of the higher transfection efficiency achieved in this muscle. We found that the expression of all three proteins, myosin heavy chain isoforms IIA/IIX, SERCA2 and ANXV, was increased in SOL muscle compared to contra-lateral control-transfected SOL muscle (Figures 3 and 4).

Examples of the immunofluorescence detection of CaMKII, MHCI and MHCII are shown in Figure 4A-C. A larger fraction of CaMKII-transfected fibres expressed MHCII than non-transfected fibres (0.36 vs. 0.18; p = 0.0008, Chi2-Test). Quantification of the co-staining of CaMKII and SERCA2 (Figure 4D/E) revealed a significantly higher staining intensity of SERCA2 in CaMKII-overexpressing SOL fibres compared to that in non-transfected fibres of the same muscle (Figure 4F).

**Figure 5:**
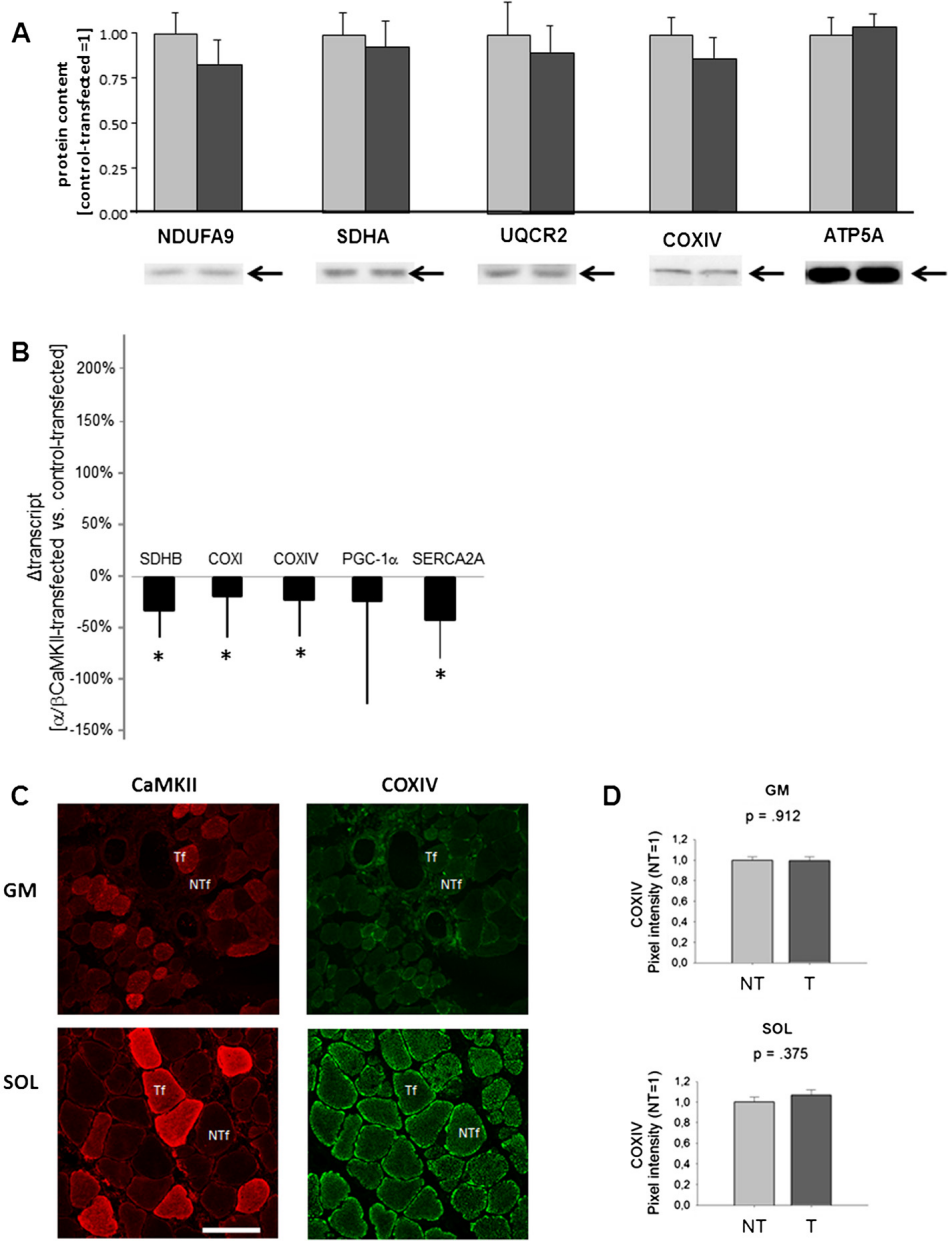
**Effect of α/β CaMKII co-overexpression on mitochondrial gene expression. A)** Bar graph visualising the mean + SE of assessed mitochondrial proteins in control-transfected (light grey filled bar) and α/β CaMKII-transfected (dark grey filled bar) SOL muscle (n = 6). Representative examples of detected mitochondrial protein are shown below. *, p ≤0.05 vs. control-transfected (Wilcoxon Test). **B)** Bar graph of mean + SE of the differences in expression of selected gene transcripts between α/β CaMKII-transfected and control-transfected SOL muscle (n = 6). * denotes p ≤0.05 vs. control-transfected muscle (T-test). **C)** Microscopic images visualising the association of the mitochondrial marker, COXIV, with transfected (Tf) and non-transfected (NTf) fibres in α/β CaMKII-transfected GM and SOL muscle. **D)** Bar graph showing the mean + SE of COXIV levels in transfected (n = 54 for GM and n = 38 for SOL) and non-transfected (n = 51 for GM and n = 35 for SOL) muscle fibres. The corresponding p-values are indicated.

Protein levels for constituents of the oxidative phosphorylation chain were not affected by α/β CaMKII overexpression (Figure 5A). We assessed whether overexpression of α/β CaMKII was sufficient to increase COXIV protein expression at the fibre level (Figure 5C). COXIV staining intensity in transfected muscles did not differ between CaMKII-overexpressing and non-transfected fibres in SOL muscle (Figure 5D).

Immunofluorescent signals in muscle fibres were also quantified in GM muscle. In this muscle, COXIV signal intensities did not differ as a function of α/β CaMKII overexpression (Figure 5D), but a larger fraction of CaMKII-transfected than non-transfected muscle fibres demonstrated MHCII staining (0.77 vs. 0.12, p = 0.048; Chi2-Test).

### CaMKII signalling after paced contractions in situ

**Figure 6:**
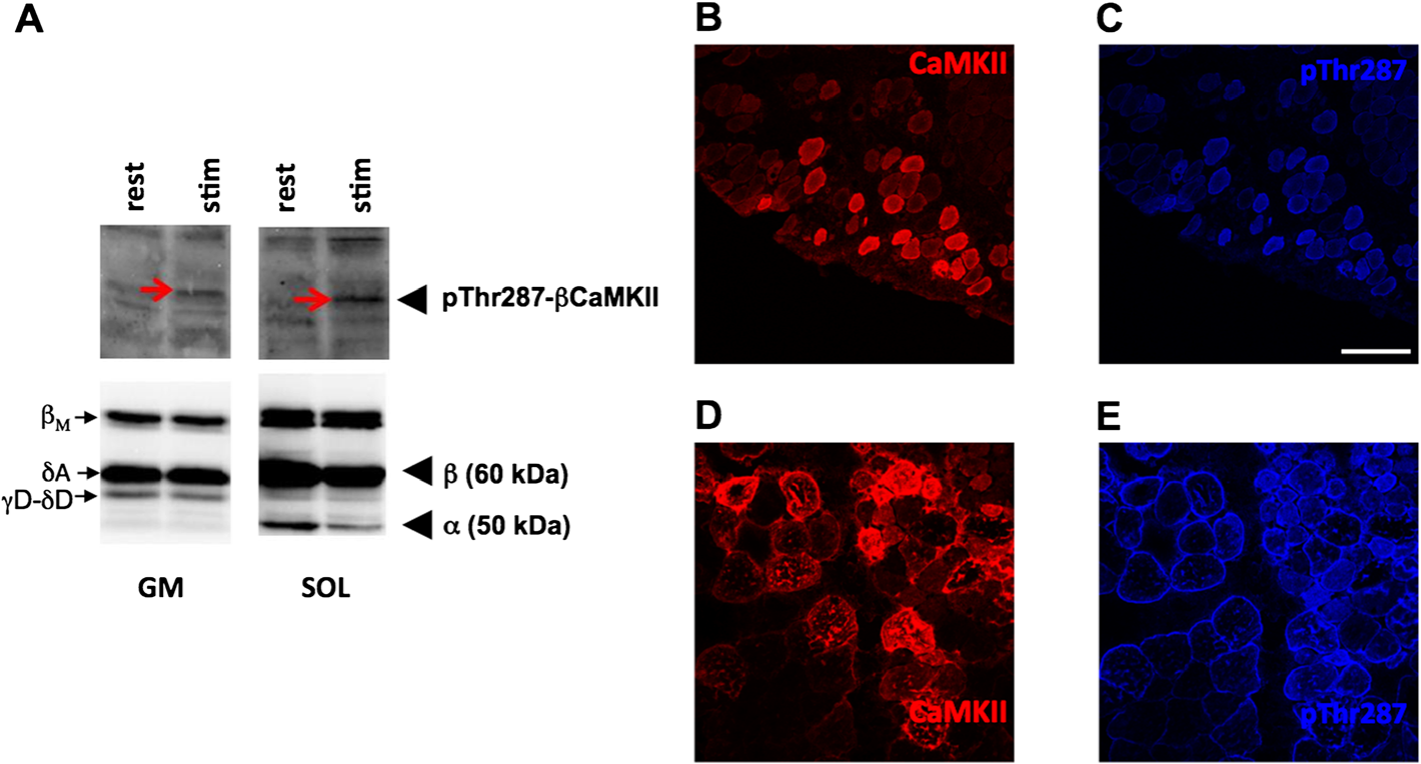
**Phosphorylation of overexpressed CaMKII after isometric exercise in situ. A)** Example of immunodetected CaMKII (top panel) and pThr287-CaMKII (bottom) in transfected GM and SOL muscle after 2-minutes of isometric exercise *in situ*. Endogenous and exogenous (bold font) CaMKII isoforms are labelled. Red arrows indicate threonine 287 phosphorylated β-CaMKII in lanes loaded with homogenate from stimulated samples (stim) compared to non-stimulated samples (rest). **B, C)** Microscopic field of α/β CaMKII-transfected SOL muscle after staining for CaMKII **(red, B)** and pThr287-CaMKII **(blue, C)**. **D, E)** Microscopic field of α/β CaMKII-transfected GM muscle after staining for CaMKII **(red, D)** and pThr287-CaMKII **(E)**. Note the congruency of CaMKII and pThr287-CaMKII stained muscle fibres. Scale bar denotes 250 μm.

We investigated whether α/β CaMKII-transfected SOL muscle retains responsiveness for contraction-induced CaMKII signalling. Paced isometric exercise *in situ* increased threonine-287 phosphorylation of β CaMKII (Figure 6A). Concomitantly, muscle fibres with elevated CaMKII content demonstrated increased signal for pThr287-CaMKII (Figure 6B-E). RT-PCR experiments demonstrated reduced transcript levels of mitochondrial factors (COXI, COXIV, SDHB) and SERCA2A immediately after high-intensity exercise (Figure 5B).

### Transfection affects muscle contractility

**Figure 7:**
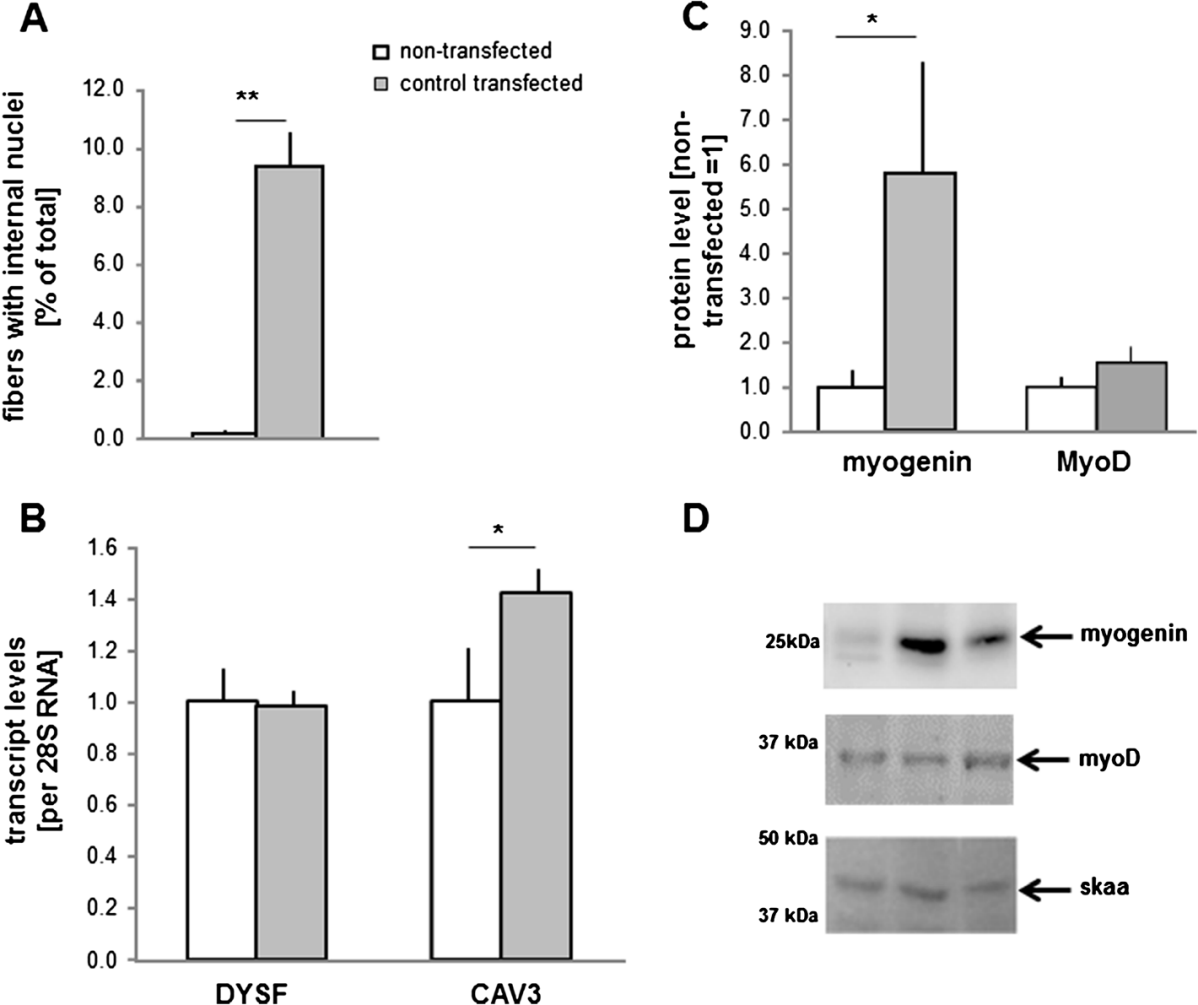
**Damage and regeneration with electroporation. A, B)** Bar graph showing mean + SE of the percentage of fibers with central nuclei **(A)** and levels of the damage markers, caveolin 3 and dysferlin mRNA, in non-transfected and control-transfected SOL muscle **(B)**. **C, D)** Bar graph of the mean + SE **(C)** and a representative immunodetection **(D)** of the myogenic marker myogenin (top), myoD (middle) and the skaa loading control (bottom) in (from left to right) non-transfected, control-transfected and a/b-transfected muscle (n=8, each). Proteins of interest are indicated with an arrow respective to the position of molecular weight markers (in kDa). * and **, p-values <0.05 and <0.001 for the indicated differences (unpaired T-test).

Compared to non-transfected muscle, twitch force (-12%) and maximal tetanic force (-30%) were reduced and time-to-peak-twitch force (+22%) and half-relaxation-time (+32%) were prolonged in control-transfected muscle (Table 1). When values were assessed separately for the two muscles, twitch force and maximum tetanic force were reduced in SOL muscle with control-transfection. Concomitantly, markers of damage (caveolin 3 mRNA) and regeneration (myogenin protein, fibres with internal nuclei) were elevated in SOL muscle 7 days after transfection (Figure 7).

**Table 1:** **Effect of α/βCaMKII-transfection vs. controls on contractile parameters**

	**SOL**			**GM**		
	***Non-transfected***	***Control-transfected***	***α/βCaMKII-transfected***	***Non-transfected***	***Control-transfected***	***α/βCaMKII-transfected***
F_twitch_ (mN)	216±9.3	178 ±15^a^	174 ±20	2548 ±86	2377 ±175^a^	2380 ±178
F_max_ (mN)	1083±57	555 ±57^a^	568 ±80	8864 ±395	7594 ±638^a^	8078 ±723
Fatigue (%)	10.5 ±3.6	18.8 ±5.3	22.7 ±5.4	67.5 ±2.1	65.3 ±2.9	67.6 ±2.2
TTP (ms)	105 ±14	133 ±7.9^a^	108 ±7.3^b^	43.0 ±3.3	50.9 ±4.0^a^	44.2 ±2.4^b^
HRT (ms)	121 ±18	168 ±4.6^a^	142 ±6.9^b^	42.9 ±5.9	54.0 ±6.0^a^	46.2 ±3.9^b^

Data are shown as mean ± SE. For abbreviations see Figure 2. Fatigue: % decrease in force after the 50 maximal tetanic contractions of the high-intensity exercise, relative to first contraction; ^a^: Main effect of electro-transfer procedure (p<0.05); ^b^: Main effect of α/βCaMKII overexpression (p<0.05).

## Discussion

This is the first study that investigates whether increased CaMKII protein expression affects the skeletal muscle phenotype *in vivo*. Transcript measures indicate that α/β CaMKII signalling down-regulates mitochondrial gene expression after high-intensity exercise (Figure 5B). In contrast to our hypothesis, we did not observe altered expression of mitochondrial protein(s) in α/β CaMKII-transfected muscle fibres of rats housed under normal cage activity (Figure 5A/D). By contrast, twitch contraction and relaxation times were reduced in α/β CaMKII-transfected muscles (Figure 2) when major proteins of muscle contraction and relaxation, i.e. MHC IIA/IIX and SERCA2, were increased (Figures 3A/B and 4). Regarding contractile features and MHCII expression in transfected muscle (fibres) the effects of α/β CaMKII overexpression were comparable in the slow-twitch and fast-twitch muscle under investigation. Our results suggest that, in the context of muscle transfection, α/β CaMKII content regulates the expression of proteins involved in muscle contraction and relaxation, but not of proteins involved in mitochondrial biogenesis.

### Technical considerations

In order to assess the effect of an increase in CaMKII holoenzymes, we deployed gene electro-transfer with expression constructs for native α- and β-CaMKII isoforms with similar substrate specificity and structure as the skeletal muscle CaMKII isoforms [35,36]. CaMKII overexpression was confined to muscle fibres (Figures 4A, 5C and 6B/D), in which CaMKII content was increased 4.0-5.3 fold compared to CaMKII content in non-transfected fibres. The latter possibly reflects the abundant β M isoform (Figure 1A; [36,41]). Immunodetection of the validated protein band for threonine 287-phosphorylated CaMKII (Figure 1C) demonstrated that the introduced β CaMKII retained responsiveness for contraction-induced CaMKII signalling (Figure 6A). The findings imply that the increased content of native CaMKII resulted in potentially higher CaMKII activity which is amenable to physiological regulation during muscle recruitment *in vivo*. It has been estimated that motor units in SOL muscles of caged, but freely moving rats are active for 25-30% during a 24-hour period [34]. Furthermore, rat SOL is recruited during postural and slow running activity [37]. Therefore, we expected transfected fibres in this muscle to be frequently recruited. We acknowledge, however, that muscle regeneration is inherent to the selected mode of transfection (Figure 7). This is of relevance for our finding that mitochondrial protein levels in α/β CaMKII-transfected SOL muscles were unaffected because muscle regeneration decreases mitochondrial enzyme expression and activity [42,43].

The identified reduction in time-to-peak-twitch force and half-relaxation time in α/β CaMKII-transfected muscles (Figure 2) support a role of CaMKII content in the regulation of excitation-contraction coupling in skeletal muscle. The comparison to non-transfected muscle reveals that this contractile effect occurred on the background of reduced contractility with transfection-induced muscle damage and regeneration (Table 1; Figure 7). Considering the broad influence of muscle damage on myogenic processes and the expression of factors involved in excitation-contraction coupling [44,45]; a number of factors are likely involved in correcting the detriment in contractility by the overexpression of α/β CaMKII. This potentially includes the calcium channels, SERCA2 and RyR, which control excitation-contraction coupling via calcium release and re-uptake in the sarcoplasmic reticulum. *In vitro* and in isolated muscle fibres both channels are regulated by CaMKII-dependent phosphorylation ([22,46], Hawkins et al., [47,48]). The characterisation of CaMKII-mediated phosphorylation was not the focus of this investigation. At present it is therefore unclear if CaMKII-dependent phosphorylation of SERCA and RyR played a role in decreasing twitch contraction and relaxation times in our experiments. Rather we identify that correction of the detriment in time-to-peak contraction and half-relaxation time by α/β CaMKII overexpression was associated with altered expression of SERCA2 and selected factors involved in excitation-contraction coupling (i.e. MHCIIA/X, ANXV). The increased SERCA2 protein levels in SOL were localised to muscle fibres that overexpress α/β CaMKII (Figure 4D-E). The localisation of SERCA2 and ANXV to the sarcoplasmic reticulum [49,50] suggests that the decrease in half-relaxation time with α/β CaMKII overexpression involves adjustments within the sarcoplasmic reticulum of transfected muscle fibres.

By contrast we observed no effect of α/β CaMKII-transfection on maximal force production (Figure 2). Regeneration has been shown to decrease specific force and increase the ratio of twitch force to tetanic force [44]. This detriment has been shown to last up to seven days after injury [43]. Therefore, it may be that any increase in maximal force was masked by negative effects of transfection on characteristics that limit force production with contraction.

Transfection of SOL muscle is associated with the expression of myosin heavy chain IIA (MHCIIA) in type I muscle fibres [39]. In this regard it is of interest that α/β CaMKII overexpression in slow-twitch SOL muscle further increased MHCIIA/X levels compared to control-transfected muscle (Figure 3A) and that the CaMKII-transfected muscle fibres demonstrated an increased fraction of MHCII expression in SOL (0.36 vs. 0.18) and GM muscle (0.77 vs. 0.12). The identified increase in SERCA2 and ANXV content with α/β CaMKII-overexpression compare to the demonstrated influence of overexpressed native CaMKII on gene expression being associated with excitation-contraction coupling and hypertrophy in cardiac myocytes [51,52]. Our findings are also in accordance with the results of Allen & Leinwand [19], who demonstrated that the calcium-ionophore A23187 increased MHCIIA promoter activity in C2C12 cells, which was attenuated by the CaMK inhibitor KN62. This raises the hypothesis that an increase in sarcoplasmic calcium levels in injured muscle fibres enhances expression of the fast fibre type program, through a CaMKII-dependent mechanism.

In contrast to our expectations, we observed no increase in mitochondrial protein (Figure 5A) and fatigue resistance (Figure 2B) in α/β CaMKII-overexpressing muscles compared to control-transfected muscles. To the best of our knowledge, this is the first study investigating whether increased content of native CaMKII increases the expression of a mitochondrial protein in any tissue type. Constitutively active CaMKIV increases mitochondrial biogenesis when overexpressed from an embryonic stage onward [18], but this CaMK is not endogenously expressed in skeletal muscle [23,32] and CaMKIV knock-out mice do not display altered muscle adaptation in response to training [23]. Whether CaMKIV would have a similar function in skeletal muscle compared to CaMKII is questionable, since the two proteins have different substrate specificity and intracellular localisation [53-55].

The results demonstrate that increased α/β CaMKII content is not sufficient to increase mitochondrial gene expression. We can therefore not rule out the possibility that CaMKII is required in conjunction with other signalling pathways for the response to exercise as shown before for the activity of a GLUT4-enhancer in mouse *m. tibialis anterior* [56]. In this regard, we identify that the reduced mitochondrial and SERCA2 transcript levels after high-intensity exercise in α/β CaMKII-transfected SOL muscle (Figure 5B) reproduce the reduced transcript expression within hour after high-load type of bicycle exercise [57] which contrasts to the up-regulation of gene transcripts with low-load endurance type exercise [58]. This suggests that effects of regeneration on mitochondrial transcript expression possibly interact with insufficient endurance type stimuli in the α/β CaMKII-transfected muscle of the rats housed under normal cage activity.

## Conclusion

Our results support a role for elevated sarcoplasmic CaMKII content for accelerating muscle contraction and relaxation in regenerating muscle via effects that include the enhanced expression of fast myosin heavy chain and sarcoplasmic reticulum associated SERCA2 and ANXV protein, but not in mitochondrial biogenesis. These observations *in vivo* are the first to point out a role for quantitative changes in this multi-functional calcium-dependent enzyme in control of the contractile muscle phenotype.

## Methods

### Experimental design

Three experimental groups from two phenotypically distinct muscles (i.e. the fast GM and slow SOL muscle) were compared in this experiment: group 1: ‘α/β *CaMKII-transfection*’, group 2: ‘*control-transfection*’, and group 3: *‘non-transfected controls*’. The comparison of group 1 vs. 2 allows identifying the feasibility and effect of CaMKII overexpression, while the comparison of groups 2 vs. 3. allows conclusions on the effects of transfection alone.

Transfection of muscle fibres was achieved via intramuscular injection of plasmid prior to the application of defined electric pulses (electro-transfer). Thereby a paired design was adopted where α/β CaMKII-transfected right SOL and GM muscles of animals were compared to the respective contralateral (i.e. left) muscles that were subjected to a control-transfection.

Effects were assessed seven days after the intervention through measurements of selected molecular, cellular and functional parameters. Functional effects were assessed *in situ* in intact muscle-tendon preparations (for abbreviations see Figure 2). The molecular measures included the quantification of the content in selected proteins using western blotting/immunodetection and the content of selected gene transcripts after high-intensity exercise *in situ*. The specificity of detecting CaMKII and threonine 287 phosphorylated CaMKII (pThr287-CaMKII) was assessed based on western blotting and immunodetection of homogenates from ‘control-transfected’ and ‘α/β CaMKII-transfected’ muscle, and homogenates which were incubated *in vitro* to assess calcium/calmodulin-dependent threonine 287 phosphorylation with commercial antibodies. The cellular specificity of CaMKII overexpression and downstream effects were assessed by quantifying immunofluorescent signals as recorded using confocal microscopy or by quantifying conventional colorimetric staining as documented with light microscopy.

### Animals

Female Wistar rats were used for the experiments described here. *In situ* contraction protocols and the majority of the electroporation experiments were carried out at the MOVE Research Institute Amsterdam, VU University Amsterdam, The Netherlands, and approved by the local committee on ethics of animal experimentation. Rat handling and experiments conformed with the Dutch Research Council’s guide for the care and use of laboratory animals. Two series of electroporation experiments were carried out at the Department of Cardiovascular Surgery, University Hospital Bern, Switzerland. These experiments were carried out according to the permission of the Animal Care Committee of the Canton of Berne (Switzerland) and following the recommendations provided by the European Convention for the protection of Vertebrate Animals used for Experimental and Scientific purposes (Strasbourg, 18.III.1986).

### Transfection

Plasmid injection followed by electropulsing was essentially carried out with modifications as described [39]. Three-month-old female Wistar rats (Harlan Laboratories/ Charles River; 191-230 grams, n = 13) were used to transfect GM and SOL muscle. Both, left and right muscles were injected with a reporter plasmid prior to electropulsing; the right muscles only were also injected with plasmid for native α and β CaMKII. pCDNA3 plasmids vectors encoding full-length cDNA for α CaMKII (pCDNA3-CaMKIIα) and β CaMKII (pCDNA3-CaMKIIβ) were a gift from Dr. M Neal Waxham (University of Texas, Houston, USA). The reporter plasmid encoding full-length luciferase under control of 424 basepairs upstream of the transcription start site of the chicken skeletal α-actin gene [59] was a gift from Dr. Frank W. Booth (University of Missouri, Columbia, USA). Animals were anaesthetized with 2-4% isoflurane through inhalation. Hindlimbs were shaved, and skin was disinfected with 70% ethanol. An incision was made into the skin and fascia between GM and *m. tibialis anterior*. SOL muscle was subsequently exposed and liberated, after which four injections of a plasmid mixture with a total volume of 90 μl were administered intramuscularly with a 29-gauge insulin syringe. Subsequently four injections of a total volume of 180 μl were administered to the GM along the length of the muscle. 5 minutes after DNA injection, the muscles were subjected to electroporation as established. 6-mm long needle electrodes (BD microlance TM3, 27G 1⁄2″; distance 4 mm) were inserted perpendicular to the fibre orientation of the injected muscle and subjected to discrete pulse protocols as established in previous experiments. For SOL the protocol involved the delivery of 3 trains of 80 × 100 microsecond pulses at 100 mA, with 992 milliseconds of interrupt on 2 locations using needle electrodes with a GET42EV generator (E.I.P. Electronique et Informatique du Pilat, Jonzieux, France) [39]. For the GM, this included the delivery of 8 trains of 60 × 100 microsecond duration at 50 mA, with 994 milliseconds interrupt on 3 locations. A mix of expression plasmid pCDNA3-CaMKIIα(0.22 μg μl^−1^) and pCDNA3-CaMKIIβ(0.22 μg μl^−1^) in TBE buffer was injected into muscles of the right leg together with the reporter plasmid (0.55 μg μl^−1^). Data obtained from this reporter construct are beyond the scope of this paper, and are therefore not reported. Muscles of the left leg were injected with the reporter plasmid only (1 μg μl^−1^). Right and left muscles of this experiment will henceforth be referred to as ‘α/β CaMKII-transfected’ and ‘control-transfected’, respectively. For the study of CaMKII activation by isometric exercise *in situ*, both left and right muscles were transfected with α/β CaMKII plasmid.

After electropulsing, the skin wound was closed with sutures, and the animal was allowed to recover from anaesthesia. Animals were kept in cages afterwards, where they resumed normal activity within hours after surgery. After seven days, animals were anaesthetized for further measurement of contraction parameters (see below) and euthanized by intra-cardiac injection of Euthasol® (VU University Amsterdam) or anaesthetized with 3% isoflurane and euthanized by dislocation of the cervical vertebrae and rapid exsanguination (University Hospital Bern). Treated muscles were harvested from both legs and snap-frozen in liquid nitrogen.

### In situ measures of muscle contraction

For measurement of isometric muscle contraction parameters, rats were anaesthetized by intra-peritoneal injections of 1.2 ml/100 gram body weight of 12.5% urethane [60]. Ear and foot reflexes were tested to check whether the animal was sufficiently anesthetized. Subsequent injections of 0.3-0.5 ml, up to a maximum of 1.5 ml, were given every 10 min afterwards until reflexes had disappeared. Experiments were carried out at room temperature (24°C). Rats were kept on a heated pad to prevent hypothermia. Hindlimbs were shaved and skin was removed, after which GM and SOL muscles were exposed and mechanically isolated by removing as much as possible the myofascial connections to surrounding muscles. Blood supply to, and nerve innervations of, both muscles were kept intact and tendons of GM and SOL muscles were attached to a force transducer via Kevlar wires. The sciatic nerve was severed proximally and connected to an external electrode that was controlled by a computer to receive pulse wave stimulation.

Optimum length of the muscle-tendon complex (the length of the muscle-tendon complex at which maximum tetanic force was produced) for isometric contractions was first estimated using twitches, then determined using a protocol consisting of two twitches and one tetanic contraction (pulse duration 100 μsec, tetanic stimulation frequency: 100 Hz, train duration: 400 ms [61]. Muscles were kept below slack length between contractions and rest duration between maximal contractions was approximately one minute. After determination of optimum length, muscles rested for five minutes. Subsequently, while muscles were set to optimum length, *a protocol of high-intensity exercise* (stimulation frequency: 100 Hz; train duration: 300 ms, one train every 800 ms, 50 trains/contractions) was applied to induce muscle fatigue. GM and SOL muscles were dissected after the end of stimulation and snap-frozen in liquid nitrogen. Animals were killed by intra-cardiac injection of Euthasol®, while still fully anaesthetized. Frozen muscles were stored at -80°C until use for western blotting/immunodetection and microscopic analysis as described below.

Force data during muscle stimulation were sampled at a frequency of 1000 Hz and collected using custom written software based on Matlab (v 7.5.0, The Mathworks Inc., MA, USA). Time-to-peak-twitch force, half-relaxation time, maximum twitch force and maximum tetanic force were determined. The same contraction parameters were determined for a group of non-transfected muscles (NT; SOL*/*GM, n = 8). Values obtained for the two twitch values in each trace were averaged.

### Isometric exercise in situ

A two-minute stimulation protocol consisting of intermittent isometric tetanic contractions of GM muscle at 100 Hz stimulation frequency [27] was applied via the sciatic nerve to α/β-CaMKII-transfected SOL muscle *in situ*. The stimulated muscles (n = 6) were freeze-clamped between liquid nitrogen-cooled aluminium grips during stimulation after two minutes. Non-stimulated, α/β-CaMKII-transfected contra-lateral muscles were subsequently dissected and frozen in liquid nitrogen. Proteins were extracted from the muscle and subjected to SDS-PAGE followed by western blotting and immunodetection as described below.

### Protein biochemistry

#### Western blotting/immunodetection

To analyse protein expression, frozen 25 μm thick cross-sections taken from the centre portion of the muscle were homogenized in ice-cold RIPA buffer (50 mM TRIS-HCl (pH 7.5), 150 mM NaCl, 1 mM EDTA, 1% v/v Nonidet P40 substitute, 0.25% w/v sodium deoxycholate) plus freshly added protease/phosphatase inhibitors: 1 mM NaF, 1 mM Na_3_VO_4_, 0.1 mM PMSF, 1 μg ml^−1^ leupeptin, 0.2 μg ml^−1^ pepstatin, 0.1 μg ml^−1^ aprotinin, using a Polytron homogenizer (Kinematica, Luzern, Switzerland). Chemicals were obtained from Sigma-Aldrich (Dorset, United Kingdom). Crude homogenates were aspirated 5-10 times through a 0.8 mm syringe needle, and stored at -80°C until use for analysis. An aliquot of the aspirated homogenate was taken for determination of protein concentration with the bicinchoninic acid protein assay (Pierce, Rockford IL, USA).

Protein levels of total CaMKII and pThr287-CaMKII were analysed by western blotting followed by immunodetection. Homogenates were denatured by addition of SDS-PAGE buffer (final concentration: 50 mM TRIS-HCl (pH 6.8), 2% w/v bromophenol blue, 10% v/v glycerol, 2% β-mercaptoethanol) and five minutes heating at 95°C. 20-40 μg of protein was separated by SDS-PAGE and transferred overnight onto a nitrocellulose membrane (GE Healthcare, Little Chalfont, UK). Membranes were stained with Ponceau S solution to confirm equal protein loading and transfer. The membrane was blocked in 5% skimmed milk in TRIS-buffered saline (pH 7.4) with 0.05% Tween-20 (TBS-T), followed by incubation with a primary antibody for pan-CaMKII (BD Bioscience #611292, dilution: 1/2500), pThr287-CaMKII (Cell Signalling Technology #3361, dilution: 1/1000), fast myosin heavy chain (Sigma-Aldrich #M4276, dilution 1/1000), myogenin (Santa Cruz Biotechnology, sc-12732 (F5D); 1:200) or myoD (Santa Cruz Biotechnology, sc-304; 1:200) or OxPhos proteins (succinate dehydrogenase Fp subunit (SDHA), ATP synthase subunit α (ATP5A), ubiquinol cytochrome c oxioreductase subunit 2 (UQCRC2) and NADH ubiquinone oxidoreductase subunit 9 (NDUFA9); #458199, Invitrogen, dilution 1/1000, and COXIV (#4850, Cell Signalling Technology, 1/2000)) for 2 hours. Antibody incubation solutions were 5% milk or 5% bovine serum albumin (BSA) in TBS-T. Finally, membranes were incubated with species-specific horseradish peroxidase-conjugated secondary antibodies (Millipore, Watford, UK). Membranes were washed in TBS-T for 4 × 5 minutes after both antibody incubations. Antibodies were detected with an enhanced chemiluminescence kit (Pierce, Rockford IL, USA). Light signals were captured with a ChemiDoc XRS system (Biorad, Hemel Hempstead, UK).

Transfected muscle pairs from the same animal were run on the same blot. Measures were limited to animals whose CaMKII injected & porated muscles showed increased expression in either of the exogenous CaMKII isoforms on a western blot. Protein bands were quantified with Quantity One version 4.6.8 (Biorad). These values were subsequently expressed as relative to the mean of the control-transfected muscles for the respective immunoblot.

### Establishing CaMKII detection

To identify CaMKII isoforms in skeletal muscle [41] buffer components were added to muscle homogenate to promote, or suppress, calcium/calmodulin dependent protein phosphorylation *in vitro* as described [62]. The reaction products were subjected to SDS-PAGE and immunoblotted for CaMKII as described in the section on ‘western blotting and immunodetection’ below. CaMKII isoforms were labelled in reference to a previous report [41]. The phosphorylated form of exogenous β CaMKII was identified based on its molecular weight (60.4 kDa), which is very similar to that of δa (60.1 kDa), the second largest CaMKII isoform in skeletal muscle [29].

### Microscopic analysis

To investigate differences in protein expression at the single fibre level, immunofluorescent staining was performed on cryosections of transfected muscles. Sections (12 μm thickness) were cut on a cryostat (CM3050, Leica, Germany) and dried for 30 minutes on glass slides. Sections were then fixed with ice-cold acetone and blocked with 5% normal goat serum in phosphate buffered saline, pH 7.5 (PBS). CaMKII and SERCA2, COXIV, MHCI or MHCII, were detected simultaneously using commercially available primary antibodies (anti-CaMKII; anti-SERCA2 (Abcam #Ab3625 or Abcam #2A7-A1, dilution 1/200); anti-COXIV (#4850, Cell Signalling Technology); anti-MHCI (#MAB1628, Millipore, dilution 1/100); anti-MHCII (#M4277, Sigma Chemicals, dilution 1/100) and species-specific Alexa 488/555 secondary antibodies (Invitrogen). Sections were washed with PBS for 4 × 5 minutes after both antibody incubations. To detect nuclei, sections were incubated for 10 minutes with TO-PRO-3 iodide (Invitrogen, Paisley, UK). Immuno-labelled sections were embedded in fluorescence compatible mounting medium (DAKO, Ely, UK).

Protein expression in electroporated fibres was analysed on a TCS SP5 confocal microscope (Leica, Milton Keynes, UK). A 10× objective was used in combination with 4× optical zoom. The fluorescent labels were excited with an Argon laser at 488 nm and HeNe lasers at 543 nm and 633 nm. The pinhole was set to match the thickness of the stained section and the focus plane was adjusted to maximize signal detection. Dyes were excited separately using a sequential scanning mode. Detected light spectra were set to maximize signal detection, but care was taken to prevent cross-excitation of dyes. Laser intensity was set to produce images with few under- or overexposed pixels, and low levels of non-specific staining, as indicated by light emission from sections that had been labelled with the secondary antibodies only. 8-Bit images were captured at 2048 × 2048 pixels, using 100 Hz scanning speed and 5-times line averaging.

The efficiency of overexpression was estimated by taking a tile scan of one cryosections of the belly portion of each α/β CaMKII-transfected muscle, which was stained for CaMKII using immunofluorescence or colorimetric staining. Subsequently individual microscopic fields were assessed for the number of fibres with prominent staining for CaMKII and the total fiber number. On average 234 and 386 fibres were counted for SOL and GM muscle, respectively. MCSA of transfected and non-transfected fibres was estimated in colorimetrically stained cross sections using Adobe Photoshop CC (Adobe Systems Incorporated) in regions of α/β CaMKII-transfected SOL muscle as described [63]. Only fibres which met the criteria of a circularity factor >0.5 were included. On average 30 transfected and 60 non-transfected fibres from the same transfected region were assessed per cross section.

SERCA2, COXIV and CaMKII staining intensity in muscle fibres overexpressing CaMKII (identified based on CaMKII staining intensity by visual inspection) was quantified with ImageJ (*rsbweb.nih.gov/ij/)*. Fibres were circumscribed manually and the average pixel intensity within the fibre was measured. An approximately equal number of fibres in the same image which did not demonstrate elevated CaMKII content (designated ‘non-transfected fibers’) was measured as well, and acted as the control group of fibres to which the CaMKII-overexpressing fibres (designated ‘transfected fibers’) were compared. MHCII expression in transfected and non-transfected fibers was assessed in the transfected region based on the presence or absence of MHC-staining as this staining was rather discrete than continuous.

Fibres with internal nuclei were assessed on 12-μm cryosections being stained with and counterstained with hematoxylin (MERCK, Germany). Microscopic files were taken at a 10-fold magnification (Axioskop 2, Carl Zeiss Ltd, Welwyn Garden City, UK) and assembled with AxioVision software (Carl Zeiss Ltd). Subsequently a grid with 250-μm unit length was superimposed and microscopic fields corresponding to 0.0625 μm2 were assessed in a random initiation, systematic manner counting fibre profiles and fibre profiles with internal (blue) nuclei. On average 475 + 50 fibre were counted per muscle.

### RT-PCR

RNA extraction from muscle tissue and RT-PCR analysis were carried out as described elsewhere [64]. Total RNA was extracted from frozen 25 μm-sections of transfected SOL muscles using the RiboPure kit (Applied Biosystems). RNA concentration and purity (260/280 nm ratio; mean: 2.06, range: 1.92-2.09) were determined using a spectrophotometer (Nanodrop Technologies, Wilmington, DE). Total RNA concentration in muscle tissue was expressed as RNA (ng) per weight of the analysed sample (mg). Five hundred nanogram of total RNA per muscle was reverse-transcribed using the high capacity RNA-to-cDNA kit (Applied Biosystems) containing random primers in a 20 μl total reaction volume. Tubes were heated at 25°C for 5 min, followed by 42°C for 30 min. Finally; the tubes were heated to 85°C for 5 min to stop the reaction and stored at -80°C until used in the PCR reaction.

For each PCR target, 5 μl of the RT reaction product was amplified in duplicate using Fast Sybr Green mastermix (Applied Biosystems). The following transcripts were targeted: 18S ribosomal RNA (18S rRNA), caveolin 3 (CAV3), dysferlin (DYSF), cytochrome-c oxidase subunit 1 (COXI), cytochrome-c oxidase 4 (COXIV), succinate dehydrogenase subunit b (SDHB), peroxisome proliferator-activated receptor γ-co-activator 1 α (PGC-1α), Annexin V (ANXV), and SERCA2A. PCR primers were designed using Primer-BLAST (http://www.ncbi.nlm.nih.gov/tools/primer-blast/). Primer sequences and Genbank accession numbers for the transcripts are shown in Table 2. Amplification efficiency of the primers used was 92.7-102.0%, and melting curve analysis demonstrated specific amplification. The range of cycle threshold values was 13-25. For all transcripts, the 18S rRNA cycle threshold was subtracted from the mean cycle threshold value of the specific target to obtain ∆C_t_ and converted into relative concentrations by 2^-∆Ct^.

**Table 2:** **Primers sequences used for RT-PCR analysis of mRNA targets**

**mRNA target mRNA**	**PCR primer sequence 5’→ 3’**	**Genbank**
18S RNA	Forward: CGAACGTCTGCCCTATCAACTT	EU 139318.1
	Reverse: ACCCGTGGTCACCATGGTA	
COXI	Forward: TGCCAGTATTAGCAGCAGGT	X14848.1
	Reverse: GAATTGGGTCTCCACCTCCA	
COXIV	Forward: AGTCCAATTGTACCGCATCC	NM 017202.1
	Reverse: ACTCATTGGTGCCCTTGTTC	
SDHB	Forward: CAGAGAAGGGATCTGTGGCT	NM 001100539.1
	Reverse: TGTTGCCTCCGTTGATGTTC	
PGC-1α	Forward: ATGAGAAGCGGGAGTCTGAA	NM 031347.1
	Reverse: GCGCTCTTCAATTGCTTTCT	
SERCA2A	Forward: GGCCCGAAACTACCTGGAGCC	NM 001110139
	Reverse: CAACGCACATGCACGCACCC	
CAV3	Forward: CCCAAGAACATCAATGAGGAC	U31968
	Reverse: GGAGACGGTGAAAGTGGTGT	
DYSF	Forward: TGGGAACCGCTACCATCTAC	NM001107869
	Reverse: CTCTGGTGCAGGAAGGAGAC	

### Statistics

Statistical analyses were carried out with STATISTICA for windows (V10.0, StatSoft, Inc. (2011), www.statsoft.com) or SPSS16 (SPSS Inc, IL, USA). One sample was identified as an outlier in the RT-PCR experiments based on the Grubb’s test and excluded from the further analysis. Immunofluorescence data from fibres demonstrating elevated or normal CaMKII levels in the same ‘α/β CaMKII-transfected’ muscle were analysed with two-tailed t-tests (unpaired). The fractions of MHCII-positive muscle fibres in the α/β CaMKII-transfected and non-transfected fibre pool, respectively, were analysed with Chi-squared tests because they represent categorical values. Western blot/immunodetection data from ‘control-transfected’ and ‘α/β CaMKII-transfected’ muscles were analysed with two-tailed Wilcoxon signed ranks tests. The effect of transfection on twitch and tetanic force parameters from ‘α/β CaMKII-transfected’ and ‘control-transfected’ muscles was tested by relating force values to the respective mean of measures from the respective ‘non-transfected muscles (i.e. GM and SOL) and running an ANOVA for the factors muscle (SOL, GM) x transfection (CaMKII-transfected, control-transfected). Non-parametric tests were run with exact significance. Significance level was set at p <0.05. Values are provided as mean ± standard error (SE).

## Abbreviations

18S rRNA: 18S ribosomal RNA
ANXV: Annexin V
ATP5A: ATP synthase H + transporting mitochondrial F1 complex
CaMKII: Calcium/calmodulin-dependent protein kinase II
CAV3: Caveolin 3
COXI: Cytochrome-c oxidase subunit 1
COXIV: Cytochrome-c oxidase subunit 4
CaM: Calmodulin
DYSF: Dysferlin
EGTA: Ethylene glycol-bis(2-aminoethylether)’-tetraacetic acid’
Ftwitch: Maximum twitch force
Fmax: Maximum tetanic force
GM: Musculus gastrocnemius
HRT: Half-relaxation time
MHCIIA: Type IIA myosin heavy chain
MHCIIB: Type IIB myosin heavy chain
MHCIIX: Type IIX myosin heavy chain
myoD: Myogenic factor 3
NDUFA9: NADH dehydrogenase (ubiquinone) 1 alpha subcomplex 9
NTf: Non-transfected
PGC-1α: Peroxisome proliferator-activated receptor γ-co-activator 1 α
PCR: Polymerase chain reaction
pThr287-CaMKII: Threonine 287 phosphorylated CaMKII
SERCA2: Sarco/endoplasmic reticulum Ca2 + -ATPase
SDHA: Succinate dehydrogenase subunit b
SDHB: Succinate dehydrogenase subunit b
SE: Standard error
skaa: Skeletal alpha actin
SOL: Musculus soleus
SR: Sarcoplasmic reticulum
Tf: Transfected
TTP: Time-to-peak-twitch force
UQCRC2: Ubiquinol-cytochrome c reductase core II protein
